# Supplementary figures and images for: Source identification of infectious diseases in networks via label ranking
Source: PLoS One. 2021 Jan 14;16(1):e0245344. doi: 10.1371/journal.pone.0245344 (PMC7808631; doi:10.1371/journal.pone.0245344)

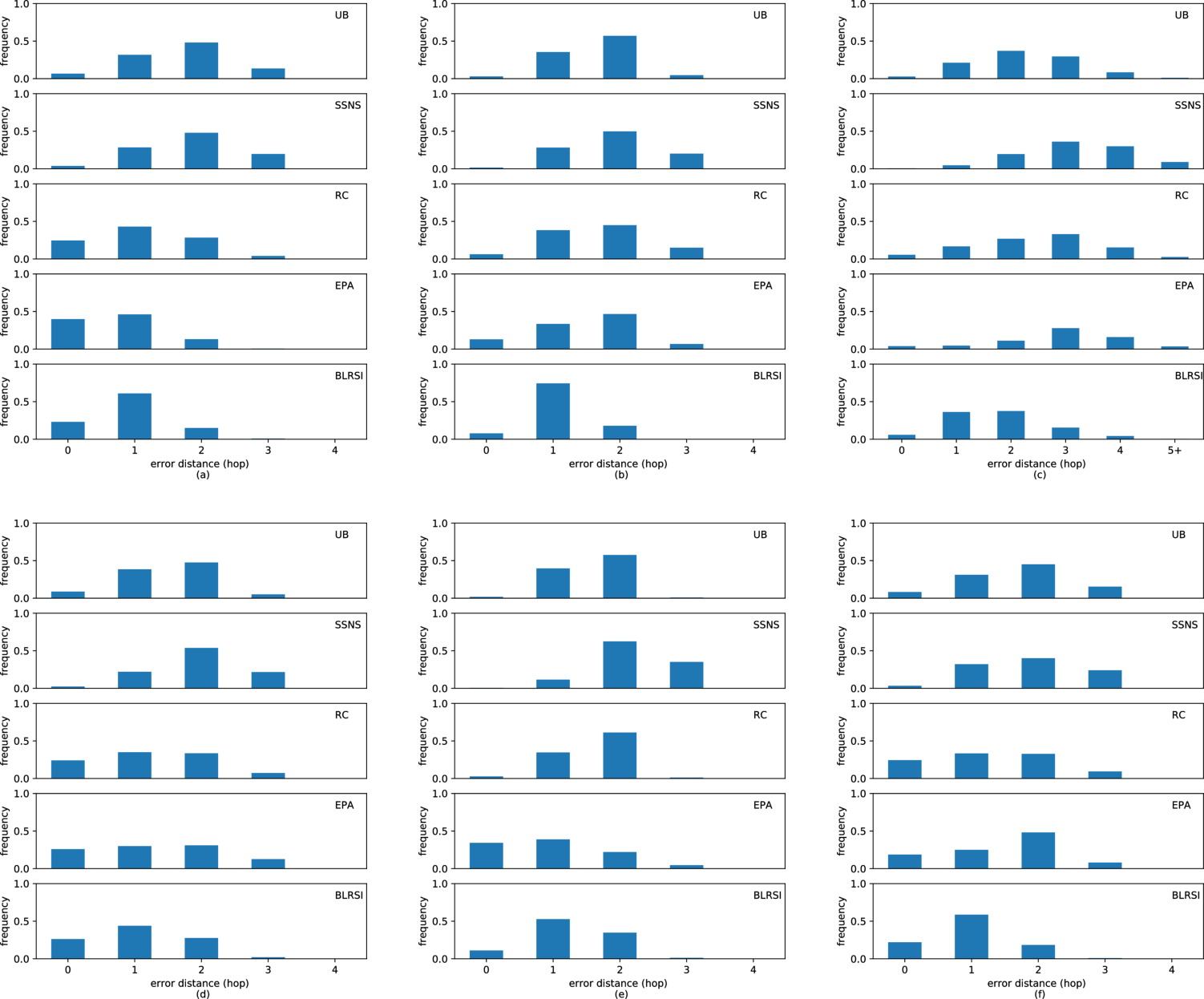

Supplement: S1 Fig — Figures (a)-(f) refer to the distributions of error distances on networks 1-6, respectively. (TIF) [file pone.0245344.s001.tif]
