# Supplementary material for: Source identification of infectious diseases in networks via label ranking
Source: PLoS One. 2021 Jan 14;16(1):e0245344. doi: 10.1371/journal.pone.0245344 (PMC7808631; doi:10.1371/journal.pone.0245344)
Supplement: Attachment — Submitted filename: Response to Reviewers.docx [file pone.0245344.s003.docx]

Dear Reviewers,

We wish to thank you for the time and effort you have spent reviewing our paper "Source Identification of Infectious Diseases in Networks via Label Ranking". We are pleased to note that you have given positive and constructive feedbacks to help us improve our work.

Motivated by your comments, we have reconsidered the details of our work and tried to improve our work. The revised manuscript has been improved significantly as follows:

1. The innovations of our work has been emphasized in the manuscript. We apply semi-supervised learning algorithms to form a label ranking framework for both complete observation and snapshot scenarios. The experimental results prove the effectiveness and efficiency of our framework.

2. Additional comparing methods are added to prove the effectiveness and efficiency of our proposed label ranking algorithms.

3. More related references and detailed discussions have been complemented.

Our itemized response to your comments, questions and suggestions (repeated below for your convenience) is as follows.

**Reviewer #1:**

**Comment 1.** About Lines 36 to 40, Lines 54 to 57, Lines 84-94 and Lines 213 to 215: There is a recent paper (Ali, S. S., et.al., EPA: Exoneration and Prominence based Age for Infection Source Identification. CIKM, Nov. 2019) which exploits and shows the importance of uninfected nodes in source identification. As, discussed in this manuscript, the paper (Ali, S.S., et al., CIKM, 2019) also talks about the prominence of a source, where a source is said to have more infected neighbor concentration than a fringe or the farthest away (from the center of an infection) node. I believe that should be added as a reference in the manuscript, besides the already included manuscript reference numbers [12] (Prakash, Aditya B., et al., ICDM, 2012) and [25] (Wang, Zheng, et al., AAAI, 2017).

**Response 1.** We have found out that the paper (Ali, S.S., et al., CIKM, 2019) would be a good complement for our work. Authors gave the theoretical explanation of the importance of uninfected nodes in source identification tasks, which supports our BLRSI algorithm. We add this paper as a reference in the manuscript.

**Comment 2.** About Lines 35 to 36: While this generally holds good, there is no mathematical proof for this. In fact, a few works show that graph centrality measures do a good job in source estimation in certain scenarios. Therefore, to inform the readers of such scenarios, besides what has already been discussed in this manuscript, I would suggest the authors add these two papers as references for the sake of unbiased dissemination of information:

1. Comin, C. H., & da Fontoura Costa, L. (2011). Identifying the starting point of a spreading process in complex networks. Physical Review E, 84(5), 056105.

2. Ali, S. S., Anwar, T., & Rizvi, S. A. M. (2020). A Revisit to the Infection Source Identification Problem under Classical Graph Centrality Measures. Online Social Networks and Media, 100061.

**Response 2.** We have mentioned (Comin, C.H., et al. Phys Rev E, 2011) as a typical source identification algorithm based on graph centrality in Lines 30 to 33. However, we believe that these graph centrality measures based on infection subgraph (which only contains infected nodes) are incomplete. Thus, we consider heuristic methods using both infected and uninfected nodes. The experimental results in the manuscript shows that our algorithm could beat centrality methods such as UB in (Comin, C.H., et al. Phys Rev E, 2011) with only infected information. The paper (Ali, S.S., et al., OSNEM, 2020) is a recent progress in centrality-based source identification algorithm and we find it valuable. We add this paper as a reference as well.

**Comment 3.** While, I don’t doubt the intentions of the authors, however, if I am not wrong, Algorithm 1 in the manuscript, i.e., BLRSI, seems starkly similar to Algorithm 1 (LPSI) of manuscript reference number [25] (Wang, Zheng, et al., AAAI, 2017). The only difference which I see is that LPSI estimates multiple sources by checking whether the label propagation score of a node with respect to its neighbors is the highest and considers it as one of the sources if that is the case. In the algorithm (BLRSI), presented in this manuscript, the highest label score amongst all the observed infected nodes is considered to be the source. I believe this has already been achieved in (Ali, S.S., et. al., A Revisit to the Infection Source Identification Problem under Classical Graph Centrality Measures. OSNEM, 2020), where, for the purpose of comparison, the authors have tuned the same algorithm (LPSI) to capture a single source in, I believe, exactly the same way as the BLRSI algorithm presented in the manuscript. Therefore, my question is what makes BLRSI algorithm different to those algorithms given BLRSI has been proposed/designed (observe the language in the Abstract Section and Line 102) in this manuscript? (Also, the analysis of BLRSI provided in Section “Algorithm analysis of Basic Label Ranking” is quite similar to the analysis of LPSI in Wang, Zheng, et al., AAAI, 2017.

**Response 3.** The intentions of our algorithm BLRSI with complete observation are indeed similar to (Wang, Zheng, et al., AAAI, 2017). We tend to measure the proportion of infected nodes surrounding each individual by BLRSI. The analysis of BLRSI is provided for the completeness of our research. Meanwhile, we discuss about the bias elimination in our analysis, which is accustomed to the source identification problem in the loss function (Comin, C.H., et al. Phys Rev E, 2011). Meanwhile, BLRSI is not the only progress of our work. We apply the label ranking in a single source scenario with both complete observation and snapshot observation and present a unified label ranking framework via the underlying semi-supervised learning methods. It makes our algorithms and framework unique among the existing approaches. We have applied several traditional semi-supervised learning loss functions (BLRSI-P1, BLRSI-P2, TSSI) to address the source identification task with complete observation and snapshot. As we mentioned in the manuscript, with the label ranking framework, more semi-supervised learning algorithms, even graph neural networks could be used to obtain the label ranking scores or restore the network status. Thus, our work, including BLRSI and other label ranking algorithms would have profound significance for propagation source identification.

**Comment 4.** For complete observation, while the comparison of BLRSI against NetSleuth/SSNS (manuscript ref. no. [12] (Prakash, Aditya B., et al., ICDM, 2012)) is fine, I suggest the comparison should be made against EPA as well (Ali, S.S., et al., CIKM, 2019), given EPA also exploits uninfected nodes to estimate the source of infection. Besides, EPA is a recent work which has been shown to beat NetSleuth/SSNS (Prakash, Aditya B., et al., ICDM, 2012) in single source infection identification scenario.

**Response 4.** Since EPA is a recent study which also makes use of uninfected nodes like we do in BLRSI and achieves quite good results, we compare our label ranking algorithms with EPA as well on the 6 datasets. Experimental results can be seen in the revised manuscript. It seems that EPA would be a powerful competitor of our proposed BLRSI. However, better performances of AED and DR on most of the networks make us believe that BLRSI is still an excellent algorithm for source identification. We discuss this in the revised manuscript.

**Comment 5.** For snapshot observation, all the three proposed methods, VLRSI, TSSI-GFHF and TSSI-LGC should and can (if I am not wrong) at least be compared with one of the recent existing works. While the authors, from Lines 368-373, do argue that the existing works have different approaches in comparison to their own methods and thus the comparison is not suitable, however, in the reasons specified by them, the authors do not consider the methods (for comparison) which plainly consider the snapshot network to estimate the source from. The direct and simple way is to compare the proposed methods (especially VLRSI) with those methods which work on SIR model, for example with Reverse Infection (RI) method (Zhu et. al., Information source identification in SIR model: A sample-path-based approach, IEEE/ACM Trans. on Networking, 2016). In case, this still is not possible, the authors should explain the same.

**Response 5.** Noted that we assume the observation vector as *O* = {*O_L_*, *O_U_*}, we could observe the exact status (no matter S/I/R or S/I) of a proportion of nodes (*O_L_*). This means, we would know the true status of some uninfected nodes as well. For those algorithms designed for SIR model, only infected (I) nodes are observed. We could not distinguish S or R in this scenario, which means no exact information would be given about any uninfected node. Also, the network restoration stage needs at least one known infected and uninfected node as well. It would be unfair to compare with the algorithms for SIR model such as Reverse Infection (RI). Thus, we could only compare VLRSI with Reverse Greedy (Luo W, Tay WP, Leng M. How to identify an infection source with limited observations. IEEE J Sel Top Signal Process, 2014) by setting all observed nodes as infected ones under SIR model (which is not completely accord with our motivation of using both infected and uninfected nodes). Still, we compare these two algorithms in the revised manuscript. But the results could not completely demonstrate the effectiveness of our proposed approach. Meanwhile, we point out that we design this algorithm under the situation that we could sample a proportion of individuals to obtain their exact status, which would be meaningful in real scenario.

**Comment 6.** I observe the average degree of the datasets used for experimentation is quite high and, hence, the diameter of these graphs would be on the smaller side and density on the higher side. Therefore, the AED might tend to appear on the good side of things. Lower average degree would mean, higher AED. This is also confirmed by the results (Table 2) achieved on Roget dataset with comparatively lower average degree tending to make AED higher. Typically, if the density of the graph is high, AED becomes small (which makes sense, since the diameter is small in the first place) and DR is not so good. This also explains why DR is so low even on ER graph (26%), when on ER graph source identification performance is generally better. I would suggest authors add density values and diameter values of each dataset in the dataset table (Table 1) and explain this relation (between graph diameter/density and AED/DR).

**Response 6.** The suggestion from the reviewer could be quite constructive. The potential relationship between the evaluation metrics of our source identification algorithms and network structures could be revealed from our experimental results. We have added related discussion in part Results and Discussions.

**Comment 7.** Generally, in how many iterations does BLRSI achieve convergence in the experimentations that authors have conducted? If there is some iteration number, the authors should add that in the manuscript to help future researchers work with an iterative process instead of a convergent one, wherever suitable.

**Response 7.** BLRSI generally takes 4 or 5 iterations to achieve convergence in our experimentations. We have added this result in part Results and Discussions of the revised manuscript.

**Comment 8.** It appears that the methods proposed in this work can estimate an infection source without having any information on the underlying model of infection propagation. Given the importance of this, I suggest the authors mention this clearly in the manuscript.

**Response 8.** Our proposed algorithms including BLRSI, VLRSI and TSSI could locate the infection source without knowing the underlying model of infection propagation as the reviewer mentioned. We have pointed out this information in the revised manuscript.

In conclusion, we have carefully considered the comments you made and make revision to our origin work. We appreciate your valuable suggestions and hope you would consider the manuscript acceptable.

Thanks,

Sincerely yours,

Biqing Huang

Professor

Dept. of Automation

Tsinghua University

Beijing 100084, China

Tel: 0086 10 6278 1655

Email: hbq@tsinghua.edu.cn
