# Supplementary material for: Source identification of infectious diseases in networks via label ranking
Source: PLoS One. 2021 Jan 14;16(1):e0245344. doi: 10.1371/journal.pone.0245344 (PMC7808631; doi:10.1371/journal.pone.0245344)
Supplement: Attachment — Submitted filename: ResponsetoReviewers.docx [file pone.0245344.s004.docx]

Dear Reviewers and Editors,

We are truly pleased for your feedbacks of our paper "Source Identification of Infectious Diseases in Networks via Label Ranking". We wish to thank you for the considerable time and effort to review our work.

We have absorbed your earnest advice and modify the details of our work. The revised manuscript has been more sophisticated and convincing by considering your comments. Our itemized response to the comments, questions and suggestions (repeated below for your convenience) are as follows.

**Reviewer #1:**

**Comment 1.** As the authors have admitted that BLRSI is indeed similar to LPSI (Wang, Zheng, et al., AAAI, 2017), they must explicitly state this in the manuscript especially when they start discussing BLRSI.

**Response 1.** We state that we were inspired by source prominence and borrow some ideas from LPSI (Wang, Zheng, et al., AAAI, 2017) while we start discussing our proposed label ranking algorithms.

**Comment 2.** The authors have done a commendable job comparing their proposed methods with EPA (Ali, S. S., et.al., EPA: Exoneration and Prominence based Age for Infection Source Identification. CIKM, Nov. 2019). However, the language in lines 421 and 422 in the revised manuscript is not appropriate. While it is noted that EPA’s performance is not as good as the proposed methods on Roget network, the authors should understand that EPA has a good/better performance on French network (on DR), besides being better in Football and BA. Therefore, the authors should remove the generalization in these lines and talk about specific details clearly while noting that EPA outperforms the proposed methods in 3 out of 6 networks (either in DR or AED or both) and provides a stiff competition to the proposed methods in ER graph (on DR), as shown in Table 2.

**Response 2.** We have removed the generalized discussion for the experimental results in Table 2 and talked about the specific performances of these algorithms. We admit that EPA may have a better performance on some of the small networks. But the stability of BLRSI on different networks, especially large-scale networks would make it a better and more practical souce identification algorithm.

**Comment 3.** The authors are advised to recheck the time complexity of UB, RC and SNSS (Table 3). As betweenness centrality of all the nodes in a graph involves calculating all the shortest paths between all the pairs of nodes, the complexity should be O(n^3) (see Floyd-Warshall algorithm). Similarly SNSS requires calculating eigenvalues from a matrix which generally has the time complexity of O(n^3). I believe RC on a general graph would have the complexity of O(n^3) as well. Authors need to check this again.

**Response 3.** According to the origin paper of SSNS (Prakash BA, et al. Spotting culprits in epidemics: How many and which ones?), the time complexity of SSNS is indeed O(m). The time cost of our experiments in Table 2 shows that SSNS is quicker than BLRSI, which has the time complexity of O(k*m). Same for RC and UB, they both have the time complexity of O(n^2). Time complexity of more source identification algorithms could be seen in Ref [10] of our manuscript.

**Comment 4.** In ref. [26] in the revised manuscript, the name of one of the authors is missing. Authors are advised to correct it. Here is the full reference: Ali SS, Anwar T, Rastogi A, Rizvi SA. EPA: Exoneration and Prominence based Age for Infection Source Identification. InProceedings of the 28th ACM International Conference on Information and Knowledge Management 2019 Nov 3 (pp. 891-900).

**Response 4.** We are truly sorry for missing one of the authors. We have corrected it in the revised manuscript.

**Comment 5.** In Supporting Information, "S1 Fig" is missing.

**Response 5.** We add the S1 Fig into our revised version.

**Reviewer #2:**

**Comment 1.** Firstly, the authors have provided Table 5 that analyzes the impact of the initial label vector y. It is seen that omitting the impact of either infected nodes or uninfected ones yields poor results. Although this particular observation is quite intuitive, the other observation where giving higher weights to uninfected nodes or retaining the original assignment like Eq.(1) produces superior results than giving higher weights to infected nodes could use some discussion. That is, why does punishing uninfected nodes give better results than crediting infected ones? This is an unexplored outcome of the experiment and can lead to interesting conversations or further analysis in future works. Basically, the authors could either provide some clarity on this end and explain the reasoning behind it, or perhaps provide a few more entries to the table through a small number of experiments showing that this is not an absolute outcome and will differ for datasets.

**Response 1.** The detailed impact of the initial assignment of label vector is an extensive problem of our study. We think that different properties of contact network and infection subgraph might lead to a different optimal label assignment. Since giving the same weight to infected and uninfected nodes could perform well, even if it’s not the best choice sometimes, we believe researchers could try this way as specific characteristics of the target source identification problem is always unclear. More concrete experiments could be conducted to assess this issue in the future.

**Comment 2.** Secondly, there is a small issue with Algorithm 2. This algorithm is almost similar to Algorithm 1 with minimal differences. In both these algorithms, k is set to be the parameter that represents the number of iterations. However, although this parameter is incremented in Algorithm 1 (Line 11), Algorithm 2 does not show it. Although it is intuitive that k will be incremented for reaching convergence, having this operation in one algorithm and not having it in another shows inconsistency and can be avoided. The same applies to Algorithm 4.

**Comment 3.** Thirdly, since Algorithm 2 is almost identical to Algorithm 1 with minor differences (one of them being the definition of the initial label vector) it seems unnecessary to repeatedly show the same operations all over again. Instead, the common algorithm (particularly the iterations) could be set as a subroutine and Algorithms BLRSI and VLRSI could separately call the common subroutine with different parameters. This would also give more insight regarding where exactly the difference between Algorithm 1 and 2 lies. Although this does not seem absolutely necessary, it is something the authors can consider.

**Response 2&3.** We find these two comments valuable and reorganize Algorithm 1 and Algorithm 2 in our revised version. This might improve the readability of our paper.

In conclusion, we have carefully reexamine our work according to your comments. We appreciate your valuable suggestions and hope you would consider the manuscript acceptable.

Thanks,

Sincerely yours,

Biqing Huang

Professor

Dept. of Automation

Tsinghua University

Beijing 100084, China

Tel: 0086 10 6278 1655

Email: hbq@tsinghua.edu.cn
